# MR-imaging pattern is not a predictor of occult atrial fibrillation in patients with cryptogenic stroke

**DOI:** 10.1007/s00415-019-09524-5

**Published:** 2019-09-11

**Authors:** C. Vollmuth, S. Stoesser, H. Neugebauer, A. Hansel, J. Dreyhaupt, A. C. Ludolph, J. Kassubek, K. Althaus

**Affiliations:** 1 Department of Neurology, University of Ulm, Oberer Eselsberg 45, 89081 Ulm, Germany; 2 Department of Neurology, University of Würzburg, Würzburg, Germany; 3 Department of Neurology, University of Bonn, Bonn, Germany; 4 Department for Psychiatry and Psychotherapy, Faculty of Medicine, Medical Center – University of Freiburg, University of Freiburg, Freiburg, Germany; 5 Institute of Epidemiology and Medical Biometry, University of Ulm, Ulm, Germany

**Keywords:** Cryptogenic stroke, Insertable cardiac monitor, Magnetic resonance imaging

## Abstract

**Background:**

To date, insertable cardiac monitors (ICMs) are the most effective method for the detection of occult atrial fibrillation (AF) in cryptogenic stroke. The overall detection rate after 12 months, however, is low and ranges between 12.4 and 33.3%, even if clinical predictors are considered. Ischemic stroke patients due to cardiogenic embolism present with particular lesion patterns. In patients with cryptogenic stroke, MR-imaging pattern may be a valuable predictor for AF.

**Methods:**

This is an MRI-based, retrospective, observational, comparative, single-center study of 104 patients who underwent ICM implantation after cryptogenic stroke. The findings were compared to a reference group with related stroke etiology, i.e., 166 patients with embolic stroke due to AF detected for the first time by long-term ECG. Lesion patterns were evaluated with regard to affected territories, distribution (cortical, lacunar, scattered), lesion volume, and lesion size (diameter of the lesion size > 20 mm).

**Results:**

The MR-imaging analysis of acute ischemic lesions yielded no association between AF and lesion size or volume, arterial vessel distribution, or the number of affected territories. There was no significant difference between the cohorts regarding ischemic patterns (cortical lesions, scattered lesions, and lacunar infarcts). An important clinical inference of our findings is that 10% (2 of 20) of cases in the ICM group in whom AF was detected had a lacunar infarct pattern. Similar results were shown in cases of ischemic stroke patients with AF detected for the first time by long-term ECG, with 10.9% (16 of 147) of them showing lacunar infarcts. The analysis of chronic MRI lesions revealed no differences between the groups in the rate of chronic lesions, arterial vessel distribution, or the number of affected territories. Left atrial size (LA size) and the presence of atrial runs in long-term ECG were independently associated with AF.

**Conclusions:**

In this MRI-based analysis of patients with cryptogenic stroke who had received ICM implantation, the detection rate of AF in patients with ICM was not related to the imaging pattern. In addition, the lacunar infarct pattern should not be an exclusion criterion for ICM insertion in patients with cryptogenic stroke. ICM insertion in patients with cryptogenic stroke should not be evaluated solely on the basis of reference to infarct patterns.

## Introduction

Ischemic stroke is among the leading causes of death and permanent disability [1]. Particularly, strokes associated with atrial fibrillation (AF) have been described as more severe than strokes due to other mechanisms [2, 3]. In addition, patients with AF have a five times higher risk of stroke than patients without AF [4], and the combination of AF plus a history of stroke carries a still higher risk of cerebrovascular incidents [5, 6]. In the case of embolic stroke due to AF, anticoagulation is an established effective treatment for secondary prophylaxis [7] and has been shown to be superior to aspirin [8]. AF is, however, frequently asymptomatic and intermittent; therefore, it may not be detected by standard ECG monitoring. When AF is absent, antiplatelet agents are recommended [9]. In approximately 25% of ischemic strokes, no mechanism can be determined [10, 11]. On the basis of the TOAST criteria, the remaining entity is termed stroke of undetermined etiology and is thus classified as cryptogenic stroke. In 2014, the case for a new clinical construct was published that claimed that these patients might have paroxysmal atrial fibrillation [11]. Several strategies for detection of paroxysmal AF were implemented: in-hospital monitoring [12], serial electrocardiography recordings [13, 14], monitoring with the use of external event or loop recorders [15-20], and continuous monitoring with insertable cardiac monitors (ICMs) [21-23]. The CRYSTAL AF study demonstrated that an ICM has a significantly higher detection rate of AF than conventional follow-up in patients with recent stroke [24]. Therein, the detection rate of AF in patients who underwent up to 36 months of continuous monitoring by an ICM was ninefold higher than in patients with standard ECG-monitoring. Furthermore, recent studies showed that higher age, left atrium dilatation, atrial runs, and a prolonged PR interval at the time of enrollment were independently associated with an increased incidence of AF in cryptogenic stroke patients [25-27]. Recently, a consensus recommendation on stratification for prolonged ECG monitoring was established [27].

The MRI pattern of embolic stroke may also be a valuable predictor of AF in patients with cryptogenic stroke. The aim of our study was to assess the particular MRI lesion patterns in patients with cryptogenic stroke. To give more weight to our findings, the results were compared to a reference group of stroke patients with cardiogenic embolism. To enclose this reference group, only patients with embolic stroke due to AF detected for the first time by long-term ECG were included.

## Methods

### Patients

Patients with a diagnosis of cryptogenic stroke or transient ischemic attack (TIA) who were admitted to the Stroke Unit at the Department of Neurology, University Hospital of Ulm, Germany, between July 2014 and June 2016 and who had undergone insertion of an ICM (Reveal LINQ; Medtronic, Minneapolis, MN, USA) for AF detection within 1 month were retrospectively identified by diagnostic code. According to the TOAST criteria, ischemic stroke or TIA was defined as cryptogenic if no specific cause was identified. Patients with cryptogenic TIA were only included if they presented with a definite cortical syndrome, such as aphasia, neglect, or homonymous hemianopsia. The imaging criteria of an embolic stroke of undetermined source (ESUS) were no inclusion criteria in our study. Patients with subcortical lesions, which measured < 2 cm in DWI were only included if they had no hints for microangiopathy as SVD in the MRI and no risk factors such as arterial hypertension or diabetes mellitus. Further patients with devasting stroke and no conceivable benefit of an ICM were not implanted.

Furthermore, all patients diagnosed with ischemic stroke or TIA due to AF detected for the first time by long-term ECG who were admitted to the Stroke Unit during the same period were identified. Collectively, all patients were divided into three groups: (ICM patients Ø AF) patients with cryptogenic stroke or TIA and insertable cardiac monitor (ICM) who remained in sinus rhythm (ICM patients with AF) patients with cryptogenic stroke or TIA and AF detected by an ICM, and (patients with AF due to long-term ECG) patients with ischemic stroke or TIA due to AF detected for the first time by long-term ECG. The principal neuroimaging modality in all patients was MRI. Approval of the study protocol was obtained from the local Ethics Committee of the University of Ulm, Germany (Reference 410/16).

### Clinical data

Clinical data at admission and discharge, including National Institutes of Health Stroke Scale (https://www.ninds.nih.gov/doctors/NIH_Stroke_Scale.pdf), were collected by analyzing the patient’s chart. Furthermore, risk factors such as arterial hypertension, congestive heart failure, thromboembolism history, hypercholesterolemia, diabetes mellitus, overweight, coronary heart disease, and history of cerebrovascular and cardiovascular diseases were determined by chart review. Consequently, the CHA2DS2-VASc-Score was calculated.

### Cardiac and vascular diagnostics

Cardiac diagnostic procedures included a 12-lead ECG at admission together with ECG monitoring henceforth for 72 h and at least one 24 h Holter ECG recording, assessed by cardiologists. Atrial fibrillation was determined by an episode of supraventricular cardiac arrhythmia that lasted more than 30 s. Episodes that lasted less than 30 s were not considered as AF. On the basis of ECG monitoring and Holter ECG, the presence of atrial runs was reviewed and approved by board-certified cardiologists. In transthoracic echocardiographic examinations, LA size was measured by M-mode in the parasternal axis in end-systole. All patients diagnosed with cryptogenic stroke underwent transesophageal echocardiography to rule out atrial septum defects. Extra- and intracranial brain-supplying arteries were assessed via Doppler- and Duplex sonography (Siemens Sequoia, Erlangen, Germany). To rule out severe atherosclerosis, ICM patients underwent CT angiography. The standard diagnostic in patients with AF due to long-term ECG did not include the analysis of atrial runs and of PFO.

### Insertable cardiac monitor and monitoring

Insertable cardiac monitors (Reveal LINQ; Medtronic, Minneapolis, MN, USA) were implanted in all patients with cryptogenic stroke within 1 month after the incident. Under local anesthesia, the insertion took place between the third and fourth intercostal positions parasternal on the left side, depending on the best ECG lead with a sufficient p-wave. Using the standard detection algorithm, atrial fibrillation, bradycardia, tachycardia, and asystole were analyzed and assessed by cardiologists. The minimum observation time was 1 year.

### Imaging studies

All patients were imaged using a clinical MR scanner (1.5 T MR scanner, Magnetom TIM Symphony, Siemens, Erlangen, Germany) equipped with a 12-channel head coil; transversal DWI with diffusion-sensitizing gradients in at least three orthogonal directions and *b* values of 0 and 1000 mm^2^/s together with transversal gradient recalled echo, T2-/T1-weighted sequences, and coronal fluid attenuated inversion recovery (FLAIR) sequences. Patients who underwent solely cranial CT or CT angiography were excluded, as were patients with MRI scans confounded by motion or other artifacts. Imaging analysis was conducted using an in-house PACS system (Centricity PACS-IW, version 3.7.3.9078, General Electric Healthcare, Milwaukee, USA). Lesions were analyzed by pattern, territory, and localization. Therefore, acute and chronic lesions were allocated to the following vascular territories: the right carotid artery, the left carotid artery, and the vertebrobasilar territory. Lesion patterns were then evaluated with regard to affected territories, distribution (cortical, lacunar, and scattered), lesion volume, and lesion size (diameter of the lesion size > 20 mm). Lesions were defined as scattered if at least two lesions were detected in one territory [27]. The volume of ischemic lesions was measured by determining the DWI-lesion size using an in-house developed volumetric software (TIFT). The extent of cerebral microangiopathy was analyzed using the age-related white matter changes (ARWMC) rating scale for MRI [28].

### Statistical analysis

Statistical analysis was performed using the Statistical Package for the Social Sciences version 24.0.0.0 (IBM SPSS Statistics, Armonk, NY, USA) and GraphPad Prism version 7 (GraphPad Software, San Diego, CA, USA). Differences in the frequency of categorical variables were analyzed using Pearson’s Chi-square test or Fisher’s exact test, depending on group size. Continuous variables were compared using the Mann–Whitney *U* test. All tests were performed two-tailed. Statistical significance was determined if the *p* value was less than 0.05. Correction of α levels for multiple testing was not used, as this was a retrospective exploratory study. Thus, all *p* values from statistical tests have to be interpreted only as hypothesis generating and not in a confirmatory sense. Regarding the MR-imaging analysis, interrater reliability using the Kappa coefficient (*κ*) was performed to determine consistency among raters [29].

## Results

A total of 276 patients were identified. Six patients without MRI scans were excluded. Thus, a total of 270 patients met the inclusion criteria, i.e., 166 patients with ischemic stroke or TIA due to AF detected for the first time by long-term ECG and 104 patients with a diagnosis of cryptogenic stroke or TIA and insertion of an ICM. Out of the 104 patients with cryptogenic stroke or TIA, AF was detected in 21 patients (20.2% of patients with cryptogenic stroke; 7.8% of all patients). The median time from ICM insertion to detection of AF was 20 days (IQR 10.0–49.0, range 0–268 days). The median follow-up time of patients who remained in sinus rhythm was 674.5 days (IQR 568.5–847.5, range 365–1109 days).

Baseline characteristics are shown in Table 1. The index event was an ischemic stroke in 242 (89.6%) patients and a transient ischemic attack in 28 (10.4%) patients. There was a male predominance in the ICM group with 68 (65.4%) male and 36 (34.6%) female patients, especially in the group of ICM patients in whom AF was detected which included 16 (76.2%) male patients. The mean age of the entire study sample was 72.2 years (±13.6 years, range 29–99). ICM patients Ø AF were on average the youngest group and significantly younger than patients of the other two groups. ICM patients in whom AF was detected were on average the second youngest group and significantly younger than patients in whom AF was detected on long-term ECG (68.9 vs. 78.7 years, p < 0.001). Patients with AF due to long-term ECG had the highest NIHSS, significantly higher than the two other groups. Regarding LA size, the data show no significant difference between ICM patients with AF and patients with AF due to long-term ECG. However, the LA was significantly smaller in ICM patients Ø AF than in the other two groups. ICM patients with AF had a higher rate of atrial runs than ICM patients Ø AF (61.9% vs. 30.1%; *p* < 0.05). The data showed no significant difference between ICM patients Ø AF and ICM patients with AF regarding the presence of a patent foramen ovale (27.7% vs. 19.0%, not significant). Patients with AF due to long-term ECG had a significantly higher CHA2DS2-Vasc Score than the other two groups (patients with AF due to long-term ECG vs. ICM patients with AF, 6.0 vs. 5.0, *p* < 0.001; patients with AF due to long-term ECG vs. ICM patients Ø AF, 6.0 vs. 3.0, *p* < 0.001). There was no significant difference in the CHA2DS2-Vasc Score between ICM patients Ø AF and ICM patients with AF.

**Table 1:** Patient baseline characteristics

	ICM patients Ø AF (*n* = 83)	*p* value	ICM patients Ø AF (*n* = 21)	*p* value	Patients with AF due to long-term ECG (*n* = 166)
Patients^c^	83 (30.7)		21 (7.8)		166 (61.5)
Demographics					
Age, years^a^	60.0 (±12.5)	**< 0.01**	68.9 (±11.0)	**< 0.001**	78.7 (±9.4)
Sex, male^c^	52 (62.7)	–	16 (76.2)	–	73 (44.0)
Stroke^c^	75 (90.4)	–	20 (95.2)	–	147 (88.6)
NIHSS at admission^b^	2.0 (1.0–4.8)	ns	1.5 (1.0–3.0)	** < 0.05**	4.0 (1.0–8.0)
Risc factors					
LA size in mm^a^	38.7 (±4.5)	**< 0.01**	42.5 (±3.9)	ns	43.3 (±7.1)
Atrial runs^c^	25 (30.1)	**< 0.05**	13 (61.9)	–	na
PFO^c^	23 (27.7)	ns	4 (19.0)	–	na
CHA2DS2-VASc-Score^b^	3.0 (3.0–5.0)	ns	5.0 (3.5–5.0)	**< 0.001**	6.0 (5.0–6.0)

Significant p-values are highlighted in bold

*ICM* insertable cardiac monitor, *AF* atrial fibrillation, *LT-ECG* long-term ECG, *ns* not significant, *na* not ascertainable

^a^Mean ± SD, two-sample *t* test

^b^Median (interquartile range), Mann–Whitney *U* test

^c^Number (%), Pearson Chi-square tests

The MR-imaging results for acute lesions are shown in Table 2. In total, the data showed no significant differences between the three cohorts. Detection of AF was not significantly associated with the volume of acute lesions, number of affected vascular territories, or distribution of acute lesions. There was no significant difference between groups regarding cortical lesions, scattered lesions, or lesions measuring more than 2.0 cm in DWI. The imaging criteria for an ESUS were met with a frequency of 82.7% in ICM patients Ø AF, 90.0% in ICM patients with AF, and 89.1% in patients with AF due to long-term ECG. Thus, subcortical lesions smaller than 2.0 cm in DWI, which are considered lacunar according to ESUS criteria, could be found at a frequency of 17.3% in ICM patients Ø AF, 10% in ICM patients with AF, and 10.9% in patients with AF due to long-term ECG.

**Table 2:** MRI results of acute ischemic lesions

	ICM patients Ø AF^d^ (*n* = 75)	*p *value	ICM patients with AF^d^ (*n* = 20)	*p *value	Patients with AF due to long-term ECG^d^ (*n* = 147)
Acute cerebral lesion^d^					
Volume in cm^3b^	1.4 (0.5–5.2)	ns	1.1 (0.3–6.5)	ns	3.5 (0.7–21.1)
Cortical lesion^c^	57 (76.0)	ns	17 (85.0)	ns	125 (85.0)
Lesion > 20 mm in DWI^c^	37 (49.3)	ns	11 (55.0)	ns	99 (67.3)
Lacunar lesion^c^	13 (17.3)	ns	2 (10.0)	ns	16 (10.9)
Scattered lesion^c^	17 (22.7)	ns	1 (5.0)	ns	26 (17.7)
ESUS criteria met^c^	62 (82.7)	ns	18 (90.0)	ns	131 (89.1)
Number of territories^b^	1.0 (1.0–1.0)	ns	1.0 (1.0–2.0)	ns	1.0 (1.0–1.0)
Lesion in 1 territory^c^	59 (78.7)	ns	13 (65.0)	ns	114 (77.6)
Lesions in 2 territories ^c^	12 (16.0)	ns	7 (35.0)	ns	27 (18.4)
Lesions in 3 territories^c^	4 (5.3)	ns	0 (0.0)	ns	6 (4.1)
Territories					
Carotic right^c^	35 (46.7)	ns	10 (50.0)	ns	51 (34.7)
Carotic left^c^	38 (50.7)	ns	13 (65.0)	ns	78 (53.1)
Vertebrobasilar^c^	21 (28.0)	ns	7 (35.0)	ns	55 (37.4)

*ICM* insertable cardiac monitor, *AF* atrial fibrillation, *LT-ECG* long-term ECG, *ESUS* embolic stroke of undetermined source, *ns* not significant

^a^Mean ± SD, two-sample *t* test

^b^Median (interquartile range), Mann–Whitney *U* test

^c^Number (%), Pearson Chi-square tests

^d^Patients with TIA excluded

Table 3 shows the MR-imaging results for chronic lesions. Patients with AF due to long-term ECG had the highest rate of chronic lesions (53.6%), followed by ICM patients Ø AF (45.8%) and ICM patients with AF (42.9%). However, the data show no significant difference between cohorts in the rate of chronic lesions, the distribution, or the number of affected territories. The presence of periventricular SVD was significantly higher in patients with AF due to long-term ECG than in the other two cohorts (patients with AF due to long-term ECG vs. ICM patients with AF, 1.0 vs. 1.0, *p* < 0.05; patients with AF due to long-term ECG vs. ICM patients Ø AF, 1.0 vs. 1.0, *p *< 0.001). The interrater reliability for MR-imaging analysis was *κ* = 0.93 (*p* < 0.05).

**Table 3:** MRI results of chronic lesions

	ICM patients Ø AF (*n* = 83)	*p* value	ICM patients with AF (*n* = 21)	*p* value	Patients with AF due to long-term ECG (*n* = 166)
Chronic cerebral lesion					
Presence of chronic lesions	38 (45.8)	ns	9 (42.9)	ns	89 (53.6)
Thereof cortical^c^	30 (78.9)	ns	6 (66.7)	ns	75 (84.3)
In total cortical^c^	30 (36.1)	ns	6 (28.6)	ns	75 (45.2)
Number of territories^b^	0.0 (0.0–1.0)	ns	0.0 (0.0–1.0)	ns	1.0 (1.0–1.0)
No lesion^c^	45 (54.2)	ns	12 (57.1)	ns	77 (46.4)
Lesion in 1 territory^c^	23 (27.7)	ns	7 (33.3)	ns	51 (30.7)
Lesions in 2 territories^c^	12 (14.5)	ns	2 (9.5)	ns	22 (13.3)
Lesions in 3 territories^c^	3 (3.6)	ns	0 (0.0)	ns	16 (9.6)
Territories					
Carotic right^c^	17 (20.5)	ns	3 (14.3)	ns	38 (22.9)
Carotic left^c^	12 (14.5)	ns	3 (14.3)	ns	41 (24.7)
Vertebrobasilar^c^	27 (32.5)	ns	5 (23.8)	ns	64 (38.6)
Periventricular SVD (Fazekas scale)					
Median (IQR)^b^	1.0 (0.0–2.0)	ns	1.0 (0.0–2.0)	**< 0.05**	1.0 (1.0–2.0)
Deep SVD (Fazekas scale)					
Median (IQR)^b^	1.0 (0.0–1.0)	ns	1.0 (0.0–1.5)	ns	1.0 (1.0–2.0)

Significant *p* values are highlighted in bold

*ICM* insertable cardiac monitor, *AF* atrial fibrillation, *LT-ECG* long-term ECG, *SVD* small vessel disease

^a^Mean ± SD, two-sample *t* test

^b^Median (interquartile range), Mann–Whitney *U* test

^c^Number (%), Pearson Chi-square tests

## Discussion

In this MRI-based retrospective, observational, comparative, single-center study of patients with cryptogenic stroke and an insertable cardiac monitor the detection rate of AF was not related to the imaging pattern. Regarding acute ischemic lesions our analysis yielded no association between AF and the lesion size, the vessel distribution, or the number of affected territories in patients with cryptogenic stroke. Furthermore, we could not identify particular MRI lesion patterns, i.e., cortical lesions, scattered lesions, and lacunar infarcts.

An important clinical inference of our findings is that the imaging criteria of an ESUS were met in only 90% of cases in the ICM group in whom AF was detected. Thus, 10% of ICM patients in whom AF was detected presented with a lacunar infarct pattern. Similar results were shown in cases of ischemic stroke patients with AF detected for the first time by long-term ECG, with 10.9% of them showing a lacunar infarct pattern. In agreement with our results, it is known that a small percentage of patients with a lacunar infarct pattern present with AF [30, 31]. Consequently, a lacunar infarct pattern in patients without risk factors for microangiopathy should not be considered as an exclusion criterion for ICM insertion in patients with cryptogenic stroke, especially because AF detection in patients with lacunar infarcts is less probable than in patients with non-lacunar infarcts [20].

The MR results of chronic lesions yielded no particular MRI lesion patterns as predictor of AF, as with acute ischemic lesions. There was no association between AF and the rate of chronic lesions, the number of affected territories, or the distribution. Ischemic stroke patients with AF detected for the first time by long-term ECG had the highest rate of periventricular SVD. Particularly, in patients with AF detected for the first time by long-term ECG, the rate of absence of periventricular SVD and deep SVD was lower than that in ICM patients. This might depend mainly on the age in the cohorts. Even the CRYSTAL AF study described a significantly higher presence of leukoaraiosis in the older cohort of ICM patients in whom AF was detected compared with the younger cohort of ICM patients who remained in sinus rhythm [25]. It is known that periventricular SVD and deep SVD are mainly associated with microangiopathy [25, 32]. However, age-related white matter changes might also be a result of lacunar infarcts due to AF, even if the extent to which AF influences age-related white matter changes is probably low.

In cardiac diagnostics, ICM patients who remained in sinus rhythm had a significantly smaller LA than the other cohorts. In contrast, dilatation of the left atrium was not significantly different between ICM patients with AF and ischemic stroke patients in whom AF was detected for the first time by long-term ECG. These findings illustrate the impact of LA size as an independent predictive factor for AF [27].

In our cohort, we could not identify a significant difference in CHA2DS2-Vasc Score between ICM patients with AF and ICM patients who remained in sinus rhythm. Recent studies used this score as a selection criterion for ICM insertion [2]. In our collective, 23.8% of the ICM patients with AF presented with CHA2DS2-Vasc Scores < 4. Therefore, we are critical using the CHA2DS2-Vasc Score as a single selection criterion for ICM insertion.

The strength of this analysis includes the imaging results using DWI with diffusion-sensitizing gradients in at least three orthogonal directions, T2-weighted sequences, and coronal fluid attenuated inversion recovery (FLAIR) sequences as imaging diagnostics for every patient. Furthermore, the imaging was reviewed by a second rater who was blinded to clinical details and the predefined hypothesis. Another strength represents the design of the study with three cohorts of stroke patients, closely linked to AF, and a detailed diagnostic work-up, particularly regarding patients with cryptogenic stroke.

One limitation of the study is the small number of ICM patients in whom AF was detected and the consecutive unequal distribution of patients in the other two collectives. Recent studies showed similar results regarding the detection rate of AF in ICM patients [1, 2, 26]. However, cardiac and vascular diagnostics, as well as the method of imaging analysis, vary between individual studies; consequently, a multicenter, retrospective study might suffer from different extents of diagnostic. To increase statistical power, a prospective study with a focus on MR imaging would be required. Similar to the CRYSTAL AF study [24], the median NIHSS at admission in our study was counted between 1.5 and 2 in the case of ICM patients. Silent AF is associated with death and permanent disability [1], so our results might be interpreted as low and a clinical preselection of ICM insertion cannot be ruled out, as it was done in the CRYSTAL AF study. However, ICM implantation in patients with devasting stroke should be discussed.

In conclusion, the imaging pattern of acute and chronic lesions did not increase the detection rate of AF in patients with ICM. We recommend that infarct patterns play a minor role in patient selection for ICM insertion. In addition, a lacunar infarct pattern is not alone associated with microangiopathy; thus, lacunar infarct pattern in patients without risk factors for microangiopathy should not be an exclusion criterion for ICM insertion. Analysis and selection of patients with cryptogenic stroke for ICM insertion should be individualized. Furthermore, we are critical of starting off-label anticoagulation in cryptogenic stroke patients solely on the basis of reference to infarct patterns.
