# Correction to: MR‑imaging pattern is not a predictor of occult atrial fibrillation in patients with cryptogenic stroke

**DOI:** 10.1007/s00415-020-09963-5

**Published:** 2020-06-15

**Authors:** Christoph Vollmuth, Sebastian Stoesser, Hermann Neugebauer, Anna Hansel, Jens Dreyhaupt, Albert Christian Ludolph, Jan Kassubek, Katharina Althaus

**Affiliations:** 1 Department of Neurology, University of Ulm, Oberer Eselsberg 45, 89081 Ulm, Germany; 2 Department of Neurology, University of Würzburg, Würzburg, Germany; 3 Department of Neurology, University of Bonn, Bonn, Germany; 4 Department for Psychiatry and Psychotherapy, Faculty of Medicine, Medical Center – University of Freiburg, University of Freiburg, Freiburg, Germany; 5 Institute of Epidemiology and Medical Biometry, University of Ulm, Ulm, Germany

## Correction to: Journal of Neurology (2019) 266:3058–3064 10.1007/s00415-019-09524-5

The original version of this article unfortunately contained a mistake. All author’s given names are in abbreviated form. The corrected names are given below.

Christoph Vollmuth **·** Sebastian Stoesser **·** Hermann Neugebauer **·** Anna Hansel **·** Jens Dreyhaupt **·** Albert Christian Ludolph **·** Jan Kassubek **·** Katharina Althaus

